# Outcomes of non-COVID-19 critically ill patients during the COVID-19 pandemic: A retrospective propensity score-matched analysis

**DOI:** 10.1007/s00508-021-01857-4

**Published:** 2021-04-19

**Authors:** Răzvan Bologheanu, Mathias Maleczek, Daniel Laxar, Oliver Kimberger

**Affiliations:** 1 Department of Anaesthesiology and General Intensive Care, Medical University of Vienna, Vienna, Austria; 2 Ludwig Boltzmann Institute for Digital Health and Patient Safety, Vienna, Austria

**Keywords:** Clinical outcomes, Mortality, Length of stay, Preparedness planning, ICU surge

## Abstract

**Background:**

Coronavirus disease 2019 (COVID-19) disrupts routine care and alters treatment pathways in every medical specialty, including intensive care medicine, which has been at the core of the pandemic response. The impact of the pandemic is inevitably not limited to patients with severe acute respiratory syndrome coronavirus 2 (SARS-CoV-2) infection and their outcomes; however, the impact of COVID-19 on intensive care has not yet been analyzed.

**Methods:**

The objective of this propensity score-matched study was to compare the clinical outcomes of non-COVID-19 critically ill patients with the outcomes of prepandemic patients. Critically ill, non-COVID-19 patients admitted to the intensive care unit (ICU) during the first wave of the pandemic were matched with patients admitted in the previous year. Mortality, length of stay, and rate of readmission were compared between the two groups after matching.

**Results:**

A total of 211 critically ill SARS-CoV‑2 negative patients admitted between 13 March 2020 and 16 May 2020 were matched to 211 controls, selected from a matching pool of 1421 eligible patients admitted to the ICU in 2019. After matching, the outcomes were not significantly different between the two groups: ICU mortality was 5.2% in 2019 and 8.5% in 2020, *p* = 0.248, while intrahospital mortality was 10.9% in 2019 and 14.2% in 2020, *p* = 0.378. The median ICU length of stay was similar in 2019: 4 days (IQR 2–6) compared to 2020: 4 days (IQR 2–7), *p* = 0.196. The rate of ICU readmission was 15.6% in 2019 and 10.9% in 2020, *p* = 0.344.

**Conclusion:**

In this retrospective single center study, mortality, ICU length of stay, and rate of ICU readmission did not differ significantly between patients admitted to the ICU during the implementation of hospital-wide COVID-19 contingency planning and patients admitted to the ICU before the pandemic.

## Introduction

### Background

Coronavirus disease (COVID-19), the acute respiratory illness caused by the severe acute respiratory syndrome coronavirus 2 (SARS-CoV-2), remains a global health concern almost 1 year after emerging [1]. To date, more than 80 million confirmed cases of infection with the novel coronavirus have been documented worldwide and more than 1.8 million deaths due to COVID-19 have been recorded [2]. The rapid spread of the disease and the high rates of intensive care unit (ICU) admission in hospitalized patients pose major challenges to healthcare systems worldwide [3–5]. To mitigate the impact of the outbreak, restrictions on public life, such as curfews and social distancing laws, have been adopted. Furthermore, healthcare systems have been reorganized to address the high number of patients: elective surgical activity has been reduced or cancelled, access to inpatient facilities has been restricted, visitor numbers have been limited, and provisional facilities designated for managing COVID-19 cases have been created [3, 6–8].

The epidemiology, clinical course, the outcomes, and the management strategies of COVID-19 are still the subject of ongoing investigations. The growing body of evidence on COVID-19 has led to a development of numerous management guidelines and new treatment options [9–11]; however, the impact of the pandemic inevitably extends beyond the patients infected with SARS-CoV‑2. Not only has an excess mortality been observed in several European countries during the first wave of the COVID-19 pandemic, but the number of excess deaths also surpassed the number of deaths attributed to COVID-19 in some countries [12–14]. Furthermore, the reduction of elective activity was accompanied by a decline in hospital admission rates for cardiovascular emergencies and an increase in intrahospital mortality [15–18].

### Objectives

The ICU capacity has been at the core of preparedness planning for COVID-19 and routine care has been disrupted by the pandemic [7, 19]; however, ICU performance and the outcomes of non-COVID-19 ICU patients during the pandemic have not been studied yet. The aim of the current study was to determine the impact of the COVID-19 pandemic and the contingency planning for widespread transmission of SARS-CoV‑2 on the clinical outcomes of critically ill patients treated in a single center in Vienna. We performed a vertical comparison of mortality, ICU readmission rates and ICU length of stay (ICU LOS) in patients treated during the implementation of COVID-19 associated measures with a propensity score-matched cohort of prepandemic ICU patients.

## Patients, material and methods

### Setting

The study was conducted at the Vienna General Hospital, a tertiary care center with 60 general ICU beds in units managed by the anesthesiology department and approximately 2500 ICU admissions per year. Ethical approval (EK Nr. 2174/2020 from 10.11.2020) was granted by the Ethics Committee of the Medical University of Vienna, Vienna, Austria, which waived the requirement to obtain informed consent.

Between 13 March 2020 and 16 May 2020, when Austria, similarly to other European countries, was experiencing a steep rise of COVID-19 cases, several measures were implemented to increase the capacity of healthcare facilities to care for a high number of hospitalized COVID-19 patients and particularly to deal with a surge in the number of critically ill patients. In our center, visits were initially forbidden and subsequently limited, while access to the hospital was generally restricted, and several activities deemed non-essential and non-urgent, such as elective surgeries and ambulatory care were cancelled or postponed. Furthermore, new facilities and repurposed units have been designated for COVID-19 patients. Finally, to reduce contact and to prevent the spread of COVID-19 between healthcare workers, a rotation system was created, while restrictions regarding the maximum volume of clinical work time were temporarily revoked.

### Study design

The aim of this study was to evaluate the ICU outcomes at the Vienna General Hospital during the first phase of the COVID-19 pandemic. Accordingly, we performed a vertical comparison between the clinical outcomes of patients admitted to the ICU while the COVID-19-associated measures were in place and the outcomes of critically ill patients from previous year. Using an electronic health records database, comprising clinical data of all patients treated in the ICU in our center, all critically ill non-COVID-19 patients admitted to the ICU during the study period as well as patients admitted in 2019, were identified. The larger matching pool was chosen to facilitate matching, based on the assumption that seasonal changes in outcomes were not relevant. The outcomes of interest were ICU and intrahospital mortality, the rate of readmission to the ICU and the ICU LOS. The outcomes were compared between the two groups after performing a nearest neighbor one-to-one propensity score matching.

### Patients

All patients admitted to the ICU during the implementation of COVID-19 associated measures, between 13 March and 16 May 2020 were included in the analysis, along with all patients admitted during 2019, as a pool of controls eligible for matching. Patients with an ICU LOS of less than 1 day or admitted to the ICU solely for postoperative monitoring, patients from the burns unit, children, COVID-19 patients, and patients missing relevant data were excluded. For patients who have been readmitted to the ICU, only the index ICU stay was included.

### Data collection and study variables

Demographic and clinical data of the patients included in the study were collected from a reporting database of electronic health records of all patients admitted to the ICU. Study variables included demographic data, time of admission and length of stay, comorbidities, primary reason for ICU admission, source of ICU admission, ICU admission diagnosis, organ support at admission (vasopressors and mechanical ventilation), limitation of therapy at admission and during the ICU stay, ICU LOS, ICU readmission, ICU and in-hospital mortality in the ICU, and simplified acute physiology score III (SAPS III) at ICU admission [20].

### Statistical analysis

Categorical data are reported as absolute number and relative frequency. Continuous variables are presented as median and interquartile range (IQR). The outcomes were compared between patients admitted to the ICU during the implementation of the COVID-19 related measures and the patients admitted in 2019. To account for a potential change in the case mix, propensity score matching was performed. The propensity score was estimated with logistic regression based on the following variables: age, sex, comorbidities (arterial hypertension, diabetes, malignant disease, heart failure, diabetes, chronic respiratory failure, chronic renal failure, chronic liver disease), SAPS III, and limitation of therapy. Based on the estimation of the propensity score, we conducted a nearest-neighbor one-to-one matching on the logit of the estimated propensity score, without replacement, stratified by admission diagnosis, in order to obtain an exact matching on this parameter [21]. Balance of covariate distribution in the matched sample was assessed by calculating the standardized means difference (SMD) for dichotomous and continuous variables [22].

Comparisons were performed using the Mann-Whitney U test for continuous variables, and the χ2 test for categorical variables, before and after matching. The outcomes were compared using the χ2 test (mortality and readmission rates), and the Mann-Whitney U test (ICU LOS), respectively. Statistical analysis was performed using the Python scientific ecosystem and the statsmodels module [23, 24]. A *p*-value of 0.05 was considered statistically significant.

## Results

A total of 390 non-COVID-19 ICU admissions between 13 March 2020 and 16 May 2020 were identified. Conversely, 2615 ICU admissions were documented in 2019. After excluding patients missing relevant data, readmissions, patients admitted to the burns unit, and patients admitted to the ICU for less than 1 day and for postoperative monitoring, 1632 patients eligible for matching (211 patients from 2020 and 1421 patients from 2019) were analyzed. Based on the estimation of the propensity score, every ICU patient admitted in 2020 during the implementation of COVID-19 related measures was matched with one ICU patient from 2019.

### Baseline characteristics

Before propensity score matching, demographics and morphometrics were similar between the two groups. The observed change in the case mix during was small. Patients admitted to the ICU in 2019 had a higher median SAPS III: 88 (IQR 74–105) in 2019 vs. 85 (IQR 70–101), were more frequently admitted from other ICUs: 86 (6.1%) vs. 4 (1.9%), and the ICU admissions were less frequently planned: 731 (51.4%) vs. 124 (58.8%). Patients admitted in 2019 were less frequently admitted from the operating room (OR), when compared with the patients from 2020: 892 (62.8%) vs. 154 (73%) and were more likely to suffer from respiratory failure: 128 (9%) vs. 7 (3.3%). The prevalence of other comorbidities did not differ significantly between the two groups. More patients were admitted for respiratory disease in 2019 compared to 2020: 91 (40.4%) vs. 4 (12.9%). The level of organ support at admission and the limitation of therapy orders were similar between the two groups (Table 1). After matching, the differences between baseline characteristics of the two groups were not statistically significant (Table 2). Furthermore, an exact matching on the admission diagnosis was performed.

**Table 1:** Baseline characteristics before matching

Characteristics	All patients 1632 (100%)	2019 1421 (87.1%)	2020 211 (12.9%)	*p*-value
**Age median years (IQR)**	61 (50–72)	62 (50–72)	61 (48–72)	0.6243^a^
**Male *n*** **(%)**	972 (59.6%)	850 (59.8%)	122 (57.8%)	0.6337^b^
**SAPS III score** **Median (IQR)**	88 (73–104)	88 (74–104)	85 (70–101)	0.0111^b^
**Reason for ICU admission**	–	–	–	0.7457^b^
*Medical n (%)*	256 (15.7%)	225(15.8%)	31 (14.7%)	–
*Surgical n (%)*	1376 (84.3%)	1196 (84.2%)	180 (85.3%)	–
**Weight** **Median (IQR)**	75 (65–88)	75 (65–89)	75 (64–85)	0.1959^a^
**Admission source *n*** **(%)**	–	–	–	0.0298^b^
*ED*	126 (7.7%)	109 (7.7%)	17 (8.1%)	–
*ICU*	90 (5.5%)	86 (6.1%)	4 (1.9%)	–
*OR*	1046 (64.1%)	892 (62.8%)	154 (73%)	–
*PACU*	84 (5.1%)	77 (5.4%)	7 (3.3%)	–
*Ward*	181 (11.1%)	162 (11.4%)	19 (9%)	–
*Other*	105 (6.4%)	95 (6.7%)	10 (4.7%)	–
**Planned *n*** **(%)**	855 (52.4%)	731 (51.4%)	124 (58.8%)	0.0556^b^
**Underlying disease *n*** **(%)**				
*Arterial hypertension*	668 (40.9%)	585 (41.2%)	83 (39.6%)	0.6672^b^
*Diabetes*	223 (13.7%)	196 (13.8%)	27 (12.8%)	0.7748^b^
*Cancer*	139 (8.5%)	117 (8.2%)	22 (10.4)	0.3509^b^
*Respiratory failure*	135 (8.3%)	128 (9%)	7 (3.3%)	0.0076^b^
*Heart failure*	213 (13.1%)	183 (12.9%)	30 (14.2%)	0.6675^b^
*Chronic kidney disease*	152 (9.3%)	128 (9%)	24 (11.4%)	0.3286^b^
*Chronic liver disease*	84 (5.1%)	75 (5.3%)	9 (4.3%)	0.6496^b^
**Organ support at admission *n*** **(%)**				
*Mechanical ventilation*	952 (58.3%)	829 (58.3%)	123 (58.3%)	0.9502^b^
*Vasopressor drugs*	963 (59%)	847 (59.6%)	116 (55%)	0.2297^b^
**Therapy limitation *n*** **(%)**				
*Palliative setting*	65 (4%)	57 (4%)	8 (3.8%)	0.9710^b^
*Other limitation of therapy*	120 (7.4%)	104 (7.3%)	16 (7.6%)	0.9966^b^
**Surgical diagnosis *n*** **(%)**	1376	1196	180	0.0013^b^
*Visceral surgery*	281 (20.4%)	254 (21.2%)	27 (15%)	–
*Vascular surgery*	93 (6.8%)	80 (6.7%)	13 (7.2%)	–
*Heart surgery*	127 (9.2%)	115 (9.6%)	12 (6.7%)	–
*Thoracic surgery*	72 (5.2%)	61 (5.1%)	11 (6.1%)	–
*Transplant surgery*	110 (8%)	101 (8.4%)	9 (5%)	–
*Neurosurgery*	346 (25.1%)	297 (24.8%)	49 (27.2%)	–
*Gynecology*	25 (1.8%)	16 (1.3%)	9 (5%)	–
*ENT/maxillofacial surgery*	97 (7%)	75 (6.3%)	22 (12.2%)	–
*Orthopedic surgery*	37 (2.7%)	30 (2.5%)	7 (3.9%)	–
*Trauma surgery*	118 (8.6%)	104 (8.7%)	14 (7.8%)	–
*Other*	70 (5.1%)	63 (5.3%)	7 (3.9%)	–
**Medical diagnosis *n*** **(%)**	256	225	31	0.0113^b^
*Sepsis*	17 (6.6%)	12 (5.3%)	5 (16.1%)	–
*Cardiovascular*	60 (23.4%)	51 (22.7%)	9 (29%)	–
*Respiratory*	95 (37.1%)	91 (40.4%)	4 (12.9%)	–
*Renal*	10 (3.9%)	8 (3.6%)	2 (6.5%)	–
*Neurologic*	50 (19.5%)	41 (18.2%)	9 (29%)	–
*Digestive*	16 (6.3%)	16 (7.1%)	0 (0%)	–
*Metabolic*	8 (3.1%)	6 (2.7%)	2 (6.5%)	–

Values are reported as median (*IQR* interquartile range) or *n* (%). *P*-values are calculated using Mann-Whitney U test (a) or χ2 test (b)

*SAPS III* simplified acute physiology score III, *ED* emergency department, *ICU* intensive care unit, *OR* operating room, *PACU* post-anesthesia care unit, *ENT* otorhinolaryngology

**Table 2:** Baseline characteristics after matching

Characteristics	All patients 422 (100%)	2019 211 (50%)	2020 211 (50%)	*p*-value
**Age median years (IQR)**	61 (48–71)	61 (49–71)	61 (48–72)	0.9557^a^
**Male *n*** **(%)**	256 (60.7%)	134 (63.5%)	122 (57.8%)	0.2730^b^
**SAPS III score** **Median (IQR)**	85 (72–103)	88 (74–105)	85 (70–101)	0.0641^a^
**Reason**				
*Medical n (%)*	62 (14.7%)	31 (14.7%)	31 (14.7%)	–
*Surgical n (%)*	360 (85.3%)	180 (85.3%)	180 (85.3%)	–
**Weight median kg (IQR)**	75 (65–87)	77 (66–90)	75 (64–85)	0.1081^a^
**Admission source *n*** **(%)**	–	–	–	0.2369^b^
*ED*	28 (6.6%)	11 (5.2%)	17 (8.1%)	–
*ICU*	14(3.3%)	10 (4.7%)	4 (1.9%)	–
*OR*	296 (70.1%)	142 (67.3%)	154 (73%)	–
*PACU*	17 (4%)	10(4.7%)	7 (3.3%)	–
*Ward*	40 (9.5%)	21 (10%)	19 (9%)	–
*Other*	27(6.4%)	17 (8.1%)	10 (4.7%)	–
**Planned *n*** **(%)**	246 (58.3%)	122(57.8%)	124 (58.8%)	0.9213^b^
**Underlying disease *n*** **(%)**				
*Arterial hypertension*	161 (38.2%)	78 (37%)	83 (39.3%)	0.6885^b^
*Diabetes*	52 (12.3%)	25 (11.8%)	27 (12.8%)	0.8822^b^
*Cancer*	49 (11.6%)	27 (12.8%)	22 (10.4%)	0.5433^b^
*Respiratory failure*	13 (3.1%)	6 (2.8%)	7 (3.3%)	–
*Heart failure*	48 (11.4%)	18 (8.5%)	30 (14.2%)	0.0916^b^
*Chronic kidney disease*	37 (8.8%)	13 (6.2%)	24 (11.4%)	0.0852^b^
*Chronic liver disease*	17 (4%)	8 (3.8%)	9 (4.3%)	–
**Organ support at admission *n*** **(%)**				
*Mechanical ventilation*	249 (59%)	126 (59.7%)	123 (58.3%)	0.8430^b^
*Vasopressor drugs*	239 (56.6%)	123 (58.3%)	116 (55%)	0.5556^b^
**Therapy limitation *n*** **(%)**				
*Palliative setting*	15 (3.6%)	7 (3.3%)	8 (3.8%)	–
*Other limitation of therapy*	29 (6.9%)	13 (6.2%)	16 (7.6%)	0.7003^b^
**Surgical diagnosis *n*** **(%)**	360	180	180	–
*Visceral surgery*	54 (15%)	27 (15%)	27 (15%)	–
*Vascular surgery*	26 (7.2%)	13 (7.2%)	13 (7.2%)	–
*Heart surgery*	24 (6.7%)	12 (6.7%)	12 (6.7%)	–
*Thoracic surgery*	22 (6.1%)	11 (6.1%)	11 (6.1%)	–
*Transplant surgery*	18 (5%)	9 (5%)	9 (5%)	–
*Neurosurgery*	98 (27.2%)	49 (27.2%)	49 (27.2%)	–
*Gynecology*	18 (5%)	9 (5%)	9 (5%)	–
*ENT/Maxillofacial surgery*	44 (12.2%)	22 (12.2%)	22 (12.2%)	–
*Orthopedic surgery*	14 (3.9%)	7 (3.9%)	7 (3.9%)	–
*Trauma surgery*	28 (7.8%)	14 (7.8%)	14 (7.8%)	–
*Other*	14 (3.9%)	7 (3.9%)	7 (3.9%)	–
**Medical diagnosis *n*** **(%)**	62	31	31	–
*Sepsis*	10 (16.1%)	5 (16.1%)	5 (16.1%)	–
*Cardiovascular*	18 (29%)	9 (29%)	9 (29%)	–
*Respiratory*	8 (12.9%)	4 (12.9%)	4 (12.9%)	–
*Renal*	4 (6.5%)	2 (6.5%)	2 (6.5%)	–
*Neurologic*	18 (29%)	9 (29%)	9 (29%)	–
*Digestive*	0 (0%)	0 (0%)	0 (0%)	–
*Metabolic*	4 (6.5%)	2 (6.5%)	2 (6.5%)	–

Values are reported as median (*IQR* interquartile range) or *n* (%). *P*-values are calculated using Mann-Whitney U test (a) or χ2 test (b)

*SAPS III* simplified acute physiology score III, *ED* emergency department, *ICU* intensive care unit, *OR* operating room, *PACU* post-anesthesia care unit, *ENT* otorhinolaryngology

### Outcomes

After matching, ICU and intrahospital mortality were slightly lower, albeit not significantly, in 2019 compared with 2020 during the first wave of COVID-19: ICU mortality was 5.2% (11/211 patients) in 2019, and 8.5% (18/211 patients) in 2020, *p* = 0.248, while in-hospital mortality was 10.9% (23/211 patients) in 2019, and 14.2% (30/211 patients) in 2020, *p* = 0.378. Of note, both the ICU mortality and intrahospital mortality in 2019 were higher before matching: the ICU mortality was 7.2% (103/1421 patients), and the intrahospital mortality was 12.5% (178/1421 patients). The median length of ICU stay was similar between the two groups: 4 days (IQR 2–6 days) in 2019 and 4 days (IQR 2–7 days) in 2020, *p* = 0.196. The rate of ICU readmission did not differ significantly between the two groups: in 2019, 15.6% (33/211 patients) had been readmitted to the ICU, compared to 10.9% (23/211 patients) in 2020, *p* = 0.344 (Table 3).

**Table 3:** Outcomes after propensity score matching

Outcome	All patients 422 (100%)	2019 211 (50%)	2020 211 (50%)	*p*-value
**ICU mortality ***n* (%)	29 (6.8%)	11 (5.2%)	18 (8.5%)	0. 2482^b^
**Intrahospital mortality ***n* (%)	53 (12.5%)	23 (10.9%)	30 (14.2%)	0. 3781^b^
**Readmission** *n* (%)	56 (13.3%)	33 (15.6%)	23 (10.9%)	0.1965^b^
**ICU LOS **median (IQR)	4 (2–7)	4 (2–6)	4 (2–7)	0.3449^a^

Values are reported as median (*IQR* interquartile range) or *n* (%). *P*-values are calculated using Mann-Whitney U test (a) or χ2 test (b)

*ICU* intensive care unit, *LOS* length of stay

## Discussion

The aim of this study was to evaluate the ICU outcomes during the first wave of the COVID-19 epidemic in Austria, when intensive care medicine was at the center of public health policies. To deal with the high numbers of severely ill patients requiring intensive care facilities due to COVID-19, increasing the ICU capacity, as well as judiciously making use of the available resources, was essential [7]. Furthermore, social/physical distancing measures were implemented to prevent the critical care demand from reaching ICU capacity: the justification, the timing, and the extent of these measures have been largely based on the anticipated ICU availability and occupancy [19]. The timelines of the in-hospital measures, which were imposed with slight variations in virtually all healthcare facilities, and the social distancing measures were not coincident. While a strict curfew was imposed from 16 March until 20 April, the implementation of the contingency planning was gradually reversed, and the staff rotation system was upheld until 16 May.

The impact of the pandemic and the related contingency measures on the ICU outcomes of non-COVID-19 patients has not yet been studied to our best knowledge. The COVID-19 pandemic disrupts standard care in every medical specialty, including intensive care, and shortages of essential medical resources, such as staff, drugs and ventilators, due to ICU strain beyond capacity could negatively affect the outcomes of critically ill patients [25, 26]. Furthermore, the associated preparedness planning could also negatively impact the quality of care. Involving family members in the intensive care is known to prevent delirium [27] and consequently, restricting visits could have had a negative effect [28]. Cancelling or postponing elective surgeries might have led to a change in the usual case mix and ICU acuity, which also has been associated with clinical outcomes [29]. Implementation of a rotation system with higher working hours could also have had an influence on the outcomes, as it directly influences the continuity of care and the overall volume of clinical workload [30].

Firstly, we observed a small change in the ICU case mix in the anesthesiology department. The admission from other ICUs and unplanned admissions accounted for a smaller proportion of patients admitted to the ICU during the implementation of the COVID-19 associated measures. While the proportion of patients admitted from the OR was higher in the same period, the reason for ICU admission (medical or surgical) was similar when compared to the patients admitted in 2019. These results may seem surprising, considering that elective activity had been massively restricted. We hypothesize that the increase in the proportion of planned admission is partly due to a hospital-wide decrease in acute admissions. In Austria, a decrease in the cumulative admission for certain cardiovascular emergencies has been reported [15, 31]. Similar changes have been reported in different countries [16, 17] and also different medical specialties [18, 32, 33].

We performed a vertical comparison of mortality, both in the ICU and intrahospital, readmission rates and ICU LOS in ICU patients admitted during the implementation of COVID-19 contingency phase and ICU patients admitted before the pandemic. The ICU LOS and the readmission rates were similar between the two matched groups. The ICU and intrahospital mortality were higher, although not significantly, in ICU patients admitted during the implementation of COVID-19 contingency planning, compared to patients admitted the year before in this propensity score matched study.

While our findings did not reach statistical significance, the trend of the results of this single-center study points in the same direction as the findings of other researchers, who found an increased mortality in acutely ill patients in different settings during a similar period. In Austria, the mortality in patients admitted for cardiovascular emergencies increased by 65% in a study by Bugger et al. [15]. The increase in mortality could be explained by a delay in treatment. It has been hypothesized that in the context of curfew and social distancing measures, patients had not promptly sought medical attention [33, 34]. In our study, however, most ICU admissions were planned. For the unplanned admissions, time between decision to admit and ICU arrival could not be analyzed. Since prioritization of resources, including critical care, did not occur in Austria, we believe that adequate treatment in the ICU for critically ill patients was readily available and the time to ICU admission was not longer than usual.

Altered treatment pathways and changes in the usual medical practice have also been reported. Hospital transfer time and time to intervention were found to be longer in patients with myocardial infarction during the COVID lockdown, and an increase in pharmacological reperfusion was observed [35–37]. Similarly, concerns regarding the implications of COVID-19 for the oncological patients have been raised [38, 39]. The reduction in surgical capacity resulted in delayed operations and chemotherapy or radiotherapy have been used in many centers instead. These changes in the clinical practice are expected to have a significant negative impact on the outcomes of cancer patients. While the clinical practice in the ICU in Austria did not change significantly beyond the COVID-19 preparedness planning, the impact of other factors on the outcomes of patients, such as timing of surgery and treatment before ICU admission and after discharge, cannot be assessed.

A large cohort study showed that perioperative infection with SARS-CoV‑2 was associated with high mortality, owing to respiratory complications that occurred in half of the infected patients [40]. Surgical patients had represented the majority of patients during the implementation of preparedness planning for COVID-19, similar to 2019; however, all patients who tested positive for the novel coronavirus were excluded from the analysis. Since every patient had been tested before elective surgery or upon ICU admission according to the local protocols, and additional testing was possible in patients with respiratory symptoms, the mortality in the matched sample could not have been influenced by a concomitant SARS-CoV‑2 infection.

We acknowledge several limitations of this single center study. First, mortality-based analysis provides only a global estimation of ICU performance. Several predictors of mortality, both patient-related and ICU-related, have been identified, so that the impact of the COVID-19 associated measures on mortality might be limited. Furthermore, long-term mortality is a more robust endpoint for ICU outcome measures; however, data concerning long-term out-of-hospital mortality was not available at the time of this study.

The ICU mortality can also be biased, particularly by admission policies, which due to the study design could not be accounted for. The relatively high SAPS III observed in this study due to the exclusion criteria: patients with an ICU LOS of less than 1 day and patients admitted solely for postoperative monitoring were excluded from analysis; however, during the implementation of COVID-19 contingency planning patients admitted to the ICU had lower SAPS III. Fewer patients with underlying respiratory failure were admitted, and the proportion of patients admitted from other ICUs was lower. While no definitive conclusion can be drawn, this possibly indicates a different admission policy, with fewer transfers from lower level facilities and fewer severely ill patients likely to require prolonged ventilation and intensive care, scheduled for elective surgery but also lower proportion of unplanned admissions.

Furthermore, despite using a propensity score matching design to reduce the bias due to confounding variables, we cannot exclude residual confounding. We assessed covariate balance after matching by calculating the SMD and found values higher than 0.1 for the SAPS III, age, and underlying heart failure and chronic kidney disease (Table 4). While a clear consensus on the threshold for significant imbalance is missing, this might suggest residual covariate imbalance after matching. However, the ICU and in-hospital mortality in patients admitted in 2019 were lower after matching, as it would be expected from a patient population with a higher proportion of planned admissions and lower SAPS III.

**Table 4:** Standardized differences for comparing means and prevalences (SMD) of matching variables between groups

Covariate	SMD before matching	SMD after matching
**Age**	0.048	0.006
**Male sex**	0.04	0.116
**SAPS III score**	0.168	0.155
**Underlying disease**		
*Arterial hypertension*	0.037	0.048
*Diabetes*	0.029	0.028
*Cancer*	0.075	0.074
*Respiratory failure*	0.238	0.027
*Heart failure*	0.039	0.179
*Chronic kidney disease*	0.078	0.185
*Chronic liver disease*	0.047	0.024
**Therapy limitation**		
*Palliative setting*	0.011	0.025
*Other limitation of therapy*	0.01	0.056

*SAPS III* simplified acute physiology score III

Finally, COVID-19 incidence and ICU availability, as well as the response to the pandemic, varied significantly across Europe, which may limit the external validity of the study [3, 7, 8, 19]. While the healthcare system in Austria was not completely overwhelmed and strict triage decisions were not necessary, the measures implemented to address the pandemic during its first phase reflect the best available evidence and the time constraints that influence decision-making during a public health emergency and are consistent with the European Centre for Disease Prevention and Control guidance [6]. It seems reasonable that a higher incidence and a lower ICU availability could have negatively impacted the ICU mortality, ICU LOS, and the rate of ICU readmission, as care would have deteriorated. For this reason, when managing the pandemic response, not only the ICU capacity but also the quality of care in the ICU and the clinical outcomes of non-COVID-19 patients should be taken into consideration.

## Conclusion

In this propensity score matched retrospective observational study performed in a single tertiary care center, clinical outcomes of ICU non-COVID-19 patients during the first phase of the pandemic did not differ significantly from the outcomes of patients admitted to the ICU during the previous year.
